# Using active matter to introduce spatial heterogeneity to the susceptible infected recovered model of epidemic spreading

**DOI:** 10.1038/s41598-022-15223-5

**Published:** 2022-07-04

**Authors:** P. Forgács, A. Libál, C. Reichhardt, N. Hengartner, C. J. O. Reichhardt

**Affiliations:** 1 Mathematics and Computer Science Department, Babeş-Bolyai University, Cluj-Napoca, 400084 Romania; 2 Theoretical Division and Center for Nonlinear Studies, Los Alamos National Laboratory, Los Alamos, NM 87545 USA

**Keywords:** Statistical physics, Infectious diseases, Colloids

## Abstract

The widely used susceptible-infected-recovered (S-I-R) epidemic model assumes a uniform, well-mixed population, and incorporation of spatial heterogeneities remains a major challenge. Understanding failures of the mixing assumption is important for designing effective disease mitigation approaches. We combine a run-and-tumble self-propelled active matter system with an S-I-R model to capture the effects of spatial disorder. Working in the motility-induced phase separation regime both with and without quenched disorder, we find two epidemic regimes. For low transmissibility, quenched disorder lowers the frequency of epidemics and increases their average duration. For high transmissibility, the epidemic spreads as a front and the epidemic curves are less sensitive to quenched disorder; however, within this regime it is possible for quenched disorder to enhance the contagion by creating regions of higher particle densities. We discuss how this system could be realized using artificial swimmers with mobile optical traps operated on a feedback loop.

## Introduction

Disease propagation through a heterogeneous environment has become a topic of worldwide interest. Tremendous modeling resources have been applied in efforts to control or at least predict the progress of the global pandemic. The majority of these models have as their basis the conceptually simple yet physically rich compartmentalized susceptible-infected-removed (S-I-R) representation of temporal disease evolution introduced nearly a century ago by Kermack and McKendrick^1^. Under the fundamental simplifying assumption of a mean-field, well-mixed population, in the S-I-R model the population is divided into *S* (susceptible), *I* (infected), or *R* (recovered) individuals, and the dynamic evolution of the epidemic is governed by the transition rates between these categories: a removal rate $\mu$ for transitions I $\rightarrow$ R and an infection rate that relies on the law of mass action to model transitions from $S \rightarrow I$. Individuals in a given bin are indistinguishable, and all spatial details of the system are discarded^2^. Despite their apparent simplicity, S-I-R models and their many variants provide powerful tools for forecasting the general course of an epidemic. Where these models falter is in predicting the specific course of an actual real-world epidemic. This is generally attributed to the lack of homogeneity in individual susceptibility, spatial contacts, and mixing behavior of individuals^3^ leading to stochastic effects that can not be averaged away.

Incorporation of heterogeneity has proven to be not at all straightforward, and numerous approaches have been developed over the years. For example, the population can be broken into subpopulations, each with different infection and recovery rates, or the population can be geographically subdivided into regions with diffusive terms to link to the regional S-I-R dynamics^4,5^. Additional heterogeneities in the diffusion can be achieved by incorporating patchiness into the diffusion^6,7^. Much work has been done on connecting individuals via finite-dimensional networks rather than through an infinite-dimensional mean field^2^; however, the details of the network itself make the problem even more complex since decisions must be made on what is the appropriate degree distribution for the network connectivity as well as whether the network should remain static or should be allowed to evolve either independently or in response to the progress of the disease^8,9^. The impact of heterogeneity in transmission and susceptibility is discussed in^10^.

The epidemic model with the ultimate heterogeneity treats each individual as a separate, mobile, interacting unit. Under Agent Based Modeling (ABM), also known as Individual Based Modeling^11^, heterogeneity can be included at all levels ranging from varied individual susceptibility and recovery rates, varied contacts between individuals, spatial clustering of individuals in cities or at attractive sites such as bars, and both short and long range transport of individuals such as by bus or airplane^12,13^. The flexibility of these models is also their greatest weakness, since in addition to the computational challenge of tracking potentially millions of individuals on a country-wide scale, there can be a vast number of free parameters that must be painstakingly fitted to real-world data that is not always available at the necessary resolution.

There have been surprisingly limited efforts to address a middle ground of ABM in which many but not all of the details are abstracted away to produce a model that captures spatial heterogeneity in a meaningful way without being swallowed in a proliferation of parameters. This can, in principle, be achieved either by developing more complex analytical models or simpler simulation-based models. One of the earliest approaches for simplifying simulation-based models involved cellular automata, where the mobility of individual agents could be varied up to a level consistent with the mean-field limit^14^. Individuals obeying S-I-R interactions have also been represented as moving particles that are driven and diffusing^15^, that never change direction^16^, that occasionally make long-range jumps^17^, that move at different velocities^18^, or that are confined to diffuse only within the region of their ’houses’^19^. To help mitigate the computational expense of such methods, dynamic density functional theory techniques can be applied^20^.

The significant progress made during recent years in understanding what are known as active matter models^21,22^, where individual particles are self-propelled and interact with each other on a spatial landscape that may or may not include disorder, suggests the natural step of pairing a model of S-I-R type with active particles. The active particles can be of run-and-tumble type^23^ or driven diffusive^24^. In a small system of low density, an active matter assembly was able to reproduce the mean field behavior of S-I-R^25^. Generally, however, there has been only limited work on coupling S-I-R modeling with active matter. For example, Paoluzzi et al. considered S-I-R type dynamics to examine information exchange in active clustering transitions^24^ but not aspects of the epidemic spreading itself. Recently Zhao et al. studied contagion dynamics in self-propelled flocking models and found that ordered homogeneous states reduce disease spreading while bands and clustering favor the spreading^26^.

There are a number of advantages to working with an active matter system. The well-known motility-induced phase separation (MIPS) transition from a low density gas phase to a coexistence between high and low density regions as a function of density and/or mobility of the active particles^27–30^ can provide a natural separation of the particles into clustered communities connected by disordered transport pathways. Contacts between particles can be viewed as an adaptive network that may be tuned to evolve on the same or a different time scale as the progression of the disease. Spatial heterogeneity emerges automatically in the MIPS regime, but can also be inserted using walls, traps, or obstacles. Disease dynamics in such systems can be abstracted by tracking the evolution of the number of *S*(*t*), *I*(*t*), and *R*(*t*) over time, whose temporal behavior will capture the impact of heterogeneities that are averaged out by the mean field approximations of the standard S-I-R model.

In this work, we simulate a large assembly of active matter particles in the MIPS regime where a giant cluster spontaneously emerges. We combine this model with S-I-R interactions in which all particles are initially susceptible (*S*) but can be infected with probability $\beta$ when they come into direct contact with an infected (*I*) particle. Infected particles spontaneously transition to the recovered (*R*) state at rate $\mu$, and no reinfection is allowed. We study the evolution of epidemics as the ratio of $\beta /\mu$ is varied, and consider the impact on the behavior of adding quenched disorder in the form of immobile obstacles. Increasing the number of immobile obstacles in an active matter system will increase the number of clusters and decrease their sizes. By performing large numbers of realizations, we find that inclusion of quenched disorder increases the number of “failed” outbreaks for small $\beta /\mu$ and increases the average duration of successful epidemics. When $\beta /\mu$ is sufficiently large, the system becomes insensitive to the presence of quenched disorder and approaches the mean field limit, and in this regime the epidemic propagates via spatially well-defined fronts. We also study the average number of susceptible particles surrounding an infective as a function of time, and find that this quantity is modestly altered by the addition of quenched disorder in the mean field limit of high $\beta /\mu$ but becomes strongly affected by quenched disorder as $\beta /\mu$ is reduced.

Our results indicate that for low $\beta /\mu$, the homogeneous mixing hypothesis breaks down, that is, the infection process departs from mass action and the system becomes much more sensitive to spatial quenched disorder. In the high $\beta /\mu$ regime, the mixing hypothesis is more applicable even though the epidemic is spreading via spatially localized fronts. This implies that localized epidemic mitigation efforts will be more successful at low $\beta /\mu$ but would become ineffective in higher $\beta /\mu$ regimes unless applied to the entire population.

Finally, we discuss how the system we consider could be realized experimentally using feedback control of light activated colloids, where the active behavior of the colloids can be controlled on the individual level. Experiments on this type of system have already demonstrated group formation, responsive states, and predator-prey model realizations^31–33^. There are also numerous possible ways to introduce spatial heterogeneities in active matter systems^34–39^. Techniques of this type could be used to mimic the S-I-R model with and without spatial disorder. This could permit the creation of table-top epidemic spreading models with active matter.

## Results

### Modeling and characterization of the S-I-R dynamics

We simulate $N=5000$ active particles in a two-dimensional system of size $L \times L$ where $L=200$ and where there are periodic boundary conditions in the *x* and *y* directions. The motion of the particles is obtained by integrating the following equation in discrete time:

1 $$\begin{aligned} \alpha _d {\mathbf{v}}_{i} = {\mathbf{F}}^{dd}_{i} + {\mathbf{F}}^{m}_{i} + {\mathbf{F}}^{obs}_{i}. \end{aligned}$$

**Figure 1:**
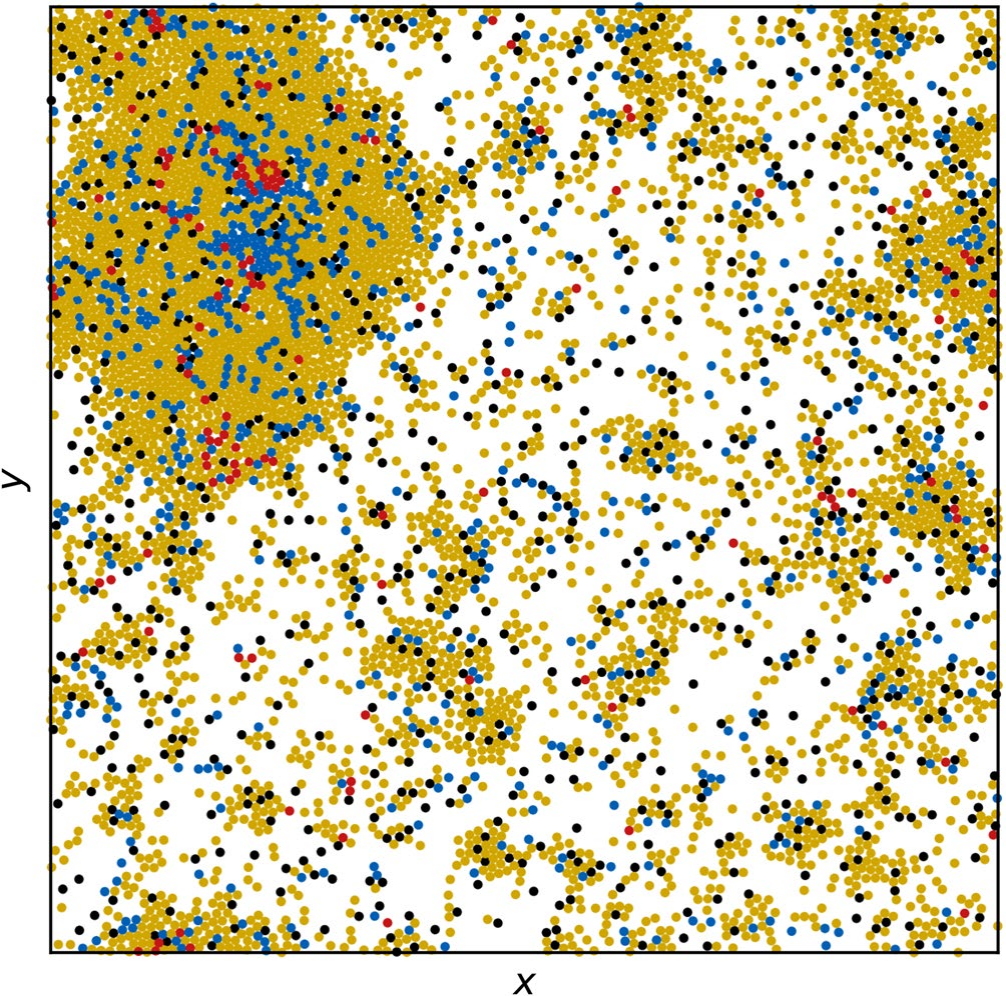
Image of the sample containing run-and-tumble S-I-R particles in a motility-induced phase separated regime. The particles transition among susceptible (*S*, yellow), infected (*I*, red), and recovered (*R*, blue) states. Here $\beta /\mu =0.5$ and quenched disorder is present in the form of $N_{\rm obs}=800$ immobile obstacles (black). The quenched disorder causes the formation of numerous small clusters in addition to the giant MIPS cluster.

Here ${\mathbf{v}}_{i} = {d {\mathbf{r}}_{i}}/{dt}$ is the velocity and ${\mathbf{r}}_{i}$ is the position of particle *i*, and the damping constant $\alpha _d = 1.0$. Time is incremented in steps of $\Delta t=0.005$. The interaction between two particles is represented with a harmonic repulsive potential ${\mathbf{F}}^{dd}_{i} = \sum _{i\ne j}^{N}k(2r_{a} - |{\mathbf{r}}_{ij}|)\Theta ( |{\mathbf{r}}_{ij}| - 2r_{a}) {\hat{\mathbf{r}}_{ij}}$, where $\Theta$ is the Heaviside step function, ${\mathbf{r}}_{ij} = {\mathbf{r}}_{i} - {\mathbf{r}}_{j}$, and $\hat{\mathbf{r}}_{ij} = {\mathbf{r}}_{ij}/|{\mathbf{r}}_{ij}|$. We set the spring force to $k = 20$ and the particle radius to $r_{a} = 1.0$. Each particle is subjected to a motor force $\mathbf{F}_i^m=F_{M}{} \mathbf{\hat{m}}_i$ of magnitude $F_M$ applied in a randomly chosen direction $\mathbf{\hat{m}}$ during a run time of $\tau _{l}$ before instantaneously changing to a new randomly chosen direction during the next run time, producing run-and-tumble dynamics. For each particle, we fix $\tau _{l}$ to a value selected randomly from the interval $1.5\times 10^4$ to $3.0 \times 10^4$ simulation time steps, and we set $F_M=1.5$ for susceptible and recovered particles, placing us in the MIPS regime in the absence of quenched disorder^37^. Infected particles have their motor force reduced to $F_M=1.0$. For some simulations, we include quenched disorder in the form of $N_{\rm obs}$ obstacles that produce the force $\mathbf{F}^{obs}$. This is taken to be the same as the particle-particle interaction force, with the only difference being that the obstacles are immobile. Unless otherwise noted, the number of obstacles is set to $N_{\rm obs}=800$. An image of the system in the presence of obstacles appears in Fig. 1.

Each active particle carries a label marking it as being in one of three states: *S*, *I*, or *R*. If an *S* particle comes into direct contact with an *I* particle, for each simulation time step during which the contact persists there is a probability $\beta$ that the *S* particle will transition to an *I* particle. If at a given simulation time step an *S* particle is in contact with *n* *I* particles, the probability of infection is $1-(1-\beta )^n \approx n\beta$. Transitions of *I* particles to state *R* occur with probability $\mu$ at each simulation time step regardless of the state of any particles that may be in contact with the *I* particle. Thus, the mean time spent in the infected state is $1/\mu$ simulation time steps. The *R* state is absorbing and *R* particles experience no further state transitions. In this S-I-R model, the infected *I* particles are present only as a transient and the system will eventually contain only *S* and/or *R* particles. We note that the mean-field rates governing $S\rightarrow I$ and $I\rightarrow R$ transitions and determining the basic reproductive number $R_0$ in classic S-I-R models do not map directly to the values of $\beta$ and $\mu$ that we insert into our model as microscopic parameters. In ABMs, the effective mean-field rates are emergent quantities instead of control parameters.

To initialize the system, we place the particles randomly in the sample and set them all to state *S*. We allow the system to evolve for $5 \times 10^5$ simulation time steps until a stable MIPS giant cluster has formed, and define this state to be the $t=0$ condition. We then randomly select 5 particles and change their state to *I*. These particles serve as our index cases, and we choose 5 rather than 1 in order to lower the probability of a failed outbreak. The system continues to evolve under both the motion of the particles and the reactions between states *S*, *I*, and *R* until no *I* particles remain. We perform 1000 realizations for each parameter set. Since, as is shown in the results, the duration $t_d = \min \{t> 0: I(t)=0\}$ of an individual epidemic can vary significantly from run to run, we report time in terms of the scaled quantity $\tilde{t}=t/t_d$. As a function of scaled time, we measure the epidemic curves $s(\tilde{t})=S(\tilde{t})/N$, $i(\tilde{t})=I(\tilde{t})/N$, and $r(\tilde{t})=R(\tilde{t})/N$. This rescaling enables us to visually compare features of the progression of the epidemic when we change the ratio $\beta /\mu$. We also measure the peak infective fraction $i_{\rm max}$ and the final susceptible fraction $s_{\infty }$, both of which are commonly used indicators of the severity of an epidemic. To obtain further information on the spatial evolution of the system, we measure the average number of susceptible particles surrounding an infective, $\eta (\tilde{t})=I^{-1}(\tilde{t})\sum _{i}^{I(\tilde{t})}\sum _{j}^{S(\tilde{t})} {\mathbb I}(|r_{ij}(\tilde{t})| = 2r_a)$, where ${\mathbb I}$ denotes the indicator function, and the sums over *i* and *j* range over the infected and susceptible particles, respectively. For a two-dimensional system of particles with identical radii $r_a$, $\eta$ cannot exceed the maximum coordination number of $z=6$. If the infected individuals are well mixed within the population, the average number of susceptible particles surrounding an infective is $\eta (\tilde{t}) \propto S(\tilde{t})$; more specifically, we would expect $\eta (\tilde{t}) \propto z_c S(\tilde{t})/N$, where $z_c$ is the average coordination number of the particles. Departure from this behavior is indicative of a failure of the homogeneous mixing assumption.

### Low transmissibility regime

**Figure 2:**
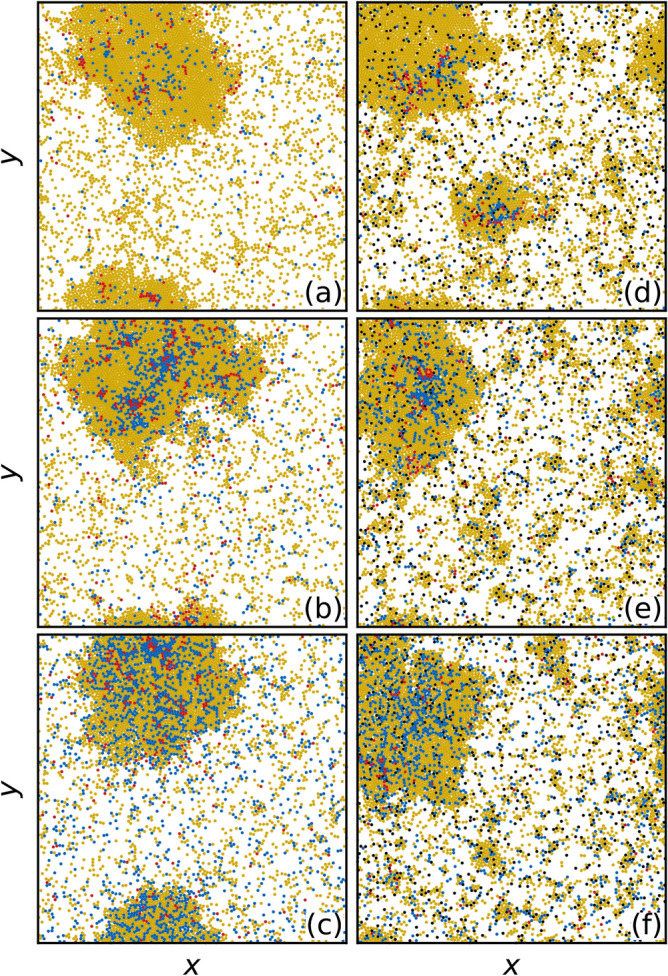
Images of the low transmissibility regime with and without quenched disorder. The evolution of the epidemic for the system in Fig. 1 with $\beta /\mu = 0.5$ at time (**a,d**) $\tilde{t}=0.2$, (**b,e**) $\tilde{t}=0.3$, and (**c,f**) $\tilde{t}=0.4$. The particles transition among susceptible (*S*, yellow), infected (*I*, red), and recovered (*R*, blue) states. (**a–c**) The obstacle-free system. (**d–f**) The system containing obstacles, showing that fewer infected particles are present at later times. Movies of these two systems are available in the Supplemental Material.

In Fig. 2a we show a snapshot of the system at $\beta /\mu =0.5$ in the low transmissibility regime in the absence of quenched disorder. The moving particles form a phase separated state of a high density solid and a low density gas. As indicated in the introduction, the relationship between $\beta /\mu$ and the basic reproductive number is an emerging quantity. Since within a cluster the expected number of contacts is $z=6$, the expected number of secondary cases from an index case within the cluster will be $\eta =3$, showing that the epidemic will infect a fraction of the cluster. If the index case starts in the gaseous phase, its expected number of contacts is likely $z<1$, implying a reproductive number less than one and giving limited cluster-to-cluster transmissions.

The same system in the presence of randomly placed obstacles appears in Fig. 2b, where the giant dense cluster is now accompanied by numerous smaller persistent clusters that have nucleated around some of the obstacle sites. Since particles within a cluster are locked to one another, the homogeneous mixing assumption fails to hold for the epidemic dynamic within each cluster. Thus by controlling the number and size of the clusters, we explore a range of departures from the mixing assumption, from the most extreme situation when there is only one large cluster, to greater mixing as the number of clusters increases and their sizes decrease.

In Fig. 2a–c we illustrate the evolution of the *S*, *I*, and *R* particles for the obstacle-free system at times of $\tilde{t}=0.2$, 0.3, and 0.4, while in Fig. 2d–f we show the evolution in the system containing $N_{\rm obs}=800$ obstacles. In both cases, when the giant cluster is contacted by an infective, the disease spreads through the cluster, but since the probability of transmission is low, not all of the *S* particles surrounding a given *I* become infected, and as a result, a finite number of *S* remain when the epidemic is complete. See Ref.^40^ for a discussion on final epidemic size. When we add quenched disorder to the system, shown as black circles in Fig. 2d–f, a greater amount of localized clustering occurs in addition to the giant cluster. Since each cluster must be infected separately, this tends to slow the spread of the infection and reduce the peak infective fraction $i_{\rm max}$, as shown in Fig. 2e.

Although the dynamics of the spread of the infection is similar with and without quenched disorder, at $\tilde{t}=0.4$ the number of *I* particles present is much lower when obstacles have broken the system into smaller clusters, indicating that the epidemic has impacted fewer particles in the system with quenched disorder.

**Figure 3:**
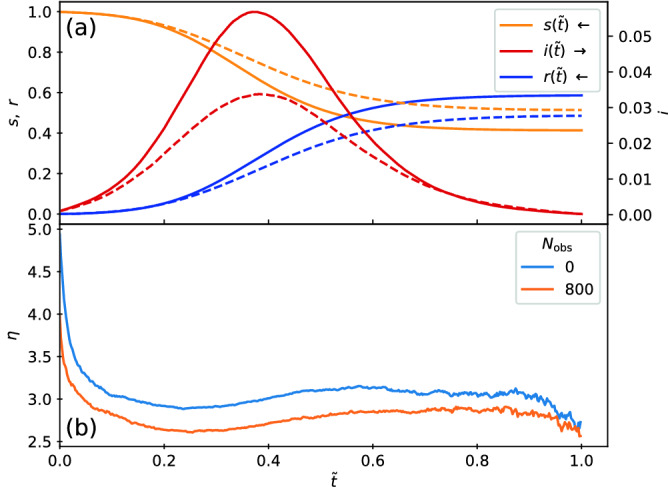
Epidemic curves in the low transmissibility regime. (**a**) Fractions of susceptible *s* (yellow), infected *i* (red), and recovered *r* (blue) particles versus rescaled time $\tilde{t}$ for the system in Fig. 2 with $\beta /\mu =0.5$. Solid lines are for samples without quenched disorder and dashed lines are for samples containing obstacles. At $\tilde{t} = 1.0$, the epidemic is over and $i=0$. Introducing obstacles reduces the peak value $i_{\rm max}$ of the infective curve. (**b**) The corresponding $\eta$, the average number of *S* particles surrounding an *I* particle, versus $\tilde{t}$ in the sample without (blue) and with (orange) obstacles. Here, the inclusion of obstacles significantly reduces $\eta$ during the entire epidemic.

In Fig. 3a we plot the epidemic curves showing the fractions of susceptible *s*, infected *i*, and recovered *r* particles as a function of reduced time $\tilde{t}$ for samples with and without quenched disorder. We note that in the presence of obstacles, the duration of the epidemic tends to be longer; however, by plotting the epidemic curves as a function of reduced time it is easier to compare samples with and without quenched disorder. The curves have the shapes expected from the classic S-I-R model. In the system without obstacles, by the end of the epidemic there is still a fraction $s_{\infty }=0.41$ of the population that never became infected, while $r_{\infty }= 1 - s_\infty =0.59$ of the particles have recovered. When obstacles are present, a larger fraction $s_{\infty }=0.51$ of particles have escaped infection. The peak $i_{\rm max}$ in the infected fraction is also considerably reduced in magnitude when obstacles are present. This indicates that the system is sensitive to the presence of spatial heterogeneities introduced by the clustering arising from the presence of fixed obstacles. Within this regime, spatially localized mitigation protocols could be effective, since local quenched disorder can slow the overall mobility of the particles or reduce the effective connectivity among the particles. To further demonstrate the effect of adding obstacles, in Fig. 3b we plot $\eta$, the average number of *S* particles surrounding an *I* particle, versus $\tilde{t}$. Here $\eta$ is always smaller in the sample containing obstacles.

**Figure 4:**
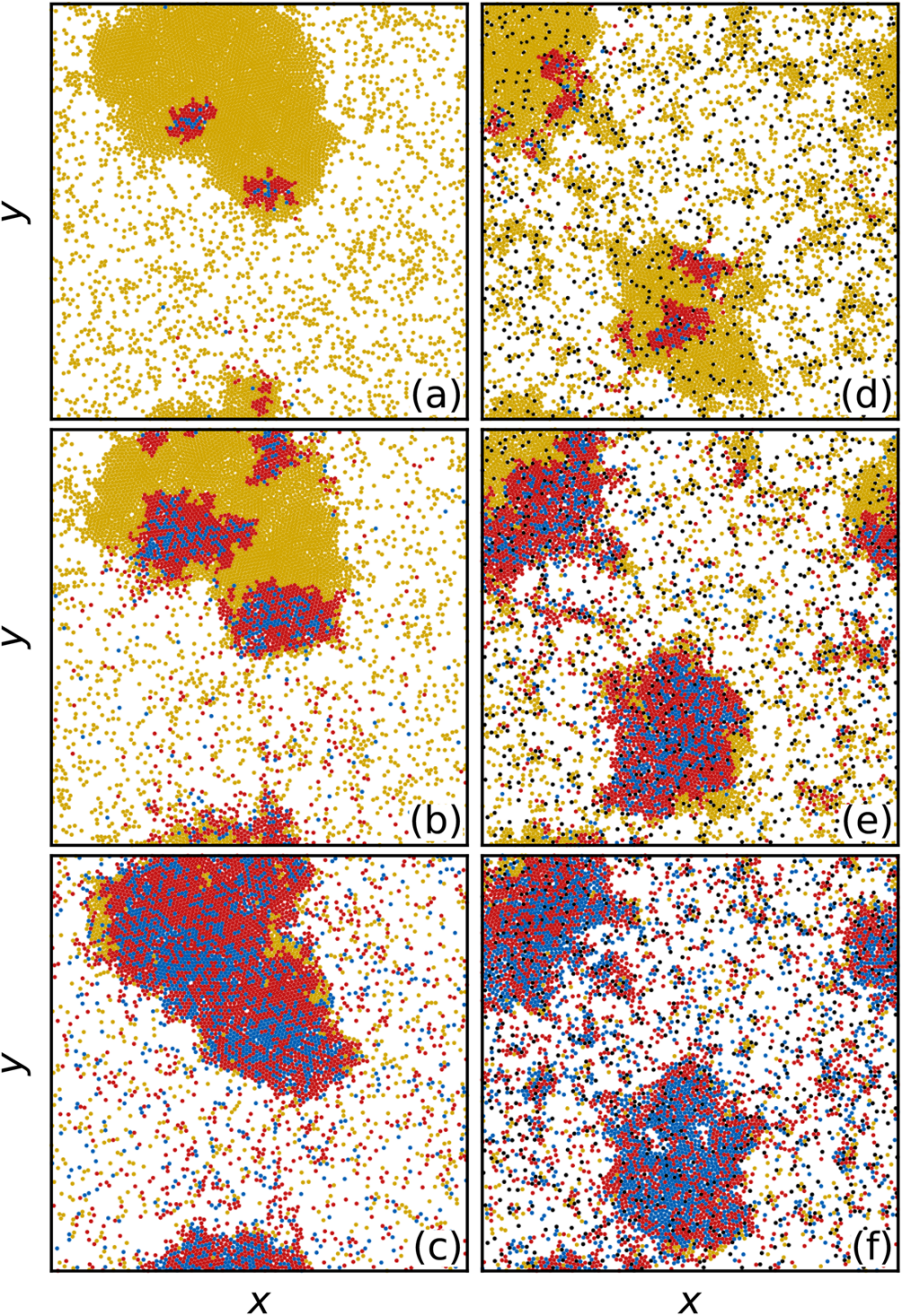
Images of the high transmissibility regime with and without quenched disorder. The evolution of the epidemic for systems with $\beta /\mu = 5.0$ at time (**a,d**) $\tilde{t}=0.1$, (**b,e**) $\tilde{t}=0.2$, and (**c,f**) $\tilde{t}=0.3$. The particles transition among susceptible (*S*, yellow), infected (*I*, red), and recovered (*R*, blue) states. (**a–c**) The obstacle-free system. (**d–f**) The system containing obstacles. For both cases, the epidemic spreads as a well-defined front through the dense clusters.

### High transmissibility regime

We next consider the case of high transmissibility $\beta /\mu =5.0$. In Fig. 4a–c we plot the spatial evolution of the susceptible, infected and recovered particles in the absence of obstacles. The infection spreads via well defined fronts through the dense region. In Fig. 4d–f we show the same evolution in the presence of obstacles. There are now multiple dense clusters present, but in each a similar front propagation of the infection appears. Movies of these two systems are available in the Supplemental Material.

**Figure 5:**
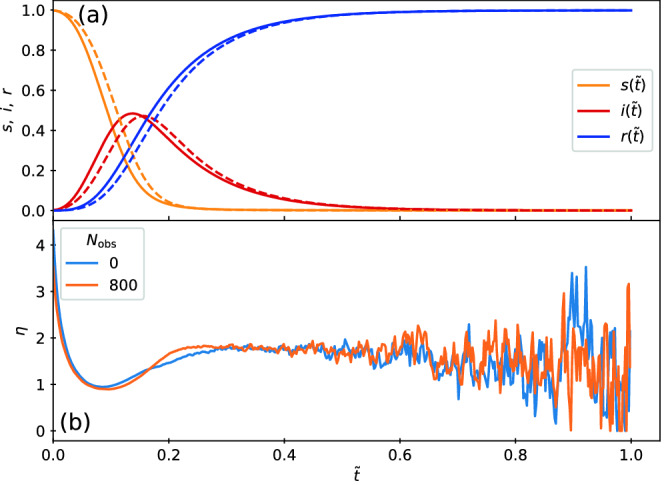
Epidemic curves in the high transmissibility regime. (**a**) Fractions of susceptible *s* (yellow), infected *i* (red), and recovered *r* (blue) particles versus reduced time $\tilde{t}$ for the system in Fig. 4 with $\beta /\mu =5.0$. Solid lines are for samples without quenched disorder and dashed lines are for samples containing obstacles. In this case all of the particles become infected and $s_{\infty }=0$. (**b**) The corresponding $\eta$, the average number of *S* particles surrounding an *I* particle, versus $\tilde{t}$ in the samples without (blue) and with (orange) obstacles. There is a minimal difference in $\eta$ between the two cases.

In Fig. 5a we plot $s(\tilde{t})$, $i(\tilde{t})$, and $r(\tilde{t})$ for the high transmissibility system with $\beta /\mu =5.0$ from Fig. 4. Here, $s_{\infty }=0$ and all of the particles become infected regardless of whether obstacles are present. The peak value $i_{\rm max}$ is nearly the same for both cases. An interesting effect appears in which for $\tilde{t} < 0.175$, adding obstacles depresses *i*, but for $\tilde{t} > 0.185$, adding obstacles increases *i*. This is not merely due to a change in the duration of the epidemic since the curves are plotted in reduced time; instead, it indicates a change in the spatial propagation of the infection, which we will address in Figs. 7 and 8. The crossover in behavior occurs after the initial large infection front has completely swept through either the giant cluster or all of the smaller clusters for the samples with quenched disorder. In Fig. 5b we plot the corresponding $\eta$ versus $\tilde{t}$, which is nearly unchanged by the inclusion of obstacles. These results indicate that under high transmissibility, the system is less sensitive to spatial disorder and the behavior is consistent with the mean field limit. Note that epidemic cuves and plots of η(t) for all other β/μ can be viewed in the Supplemental Material.

**Figure 6:**
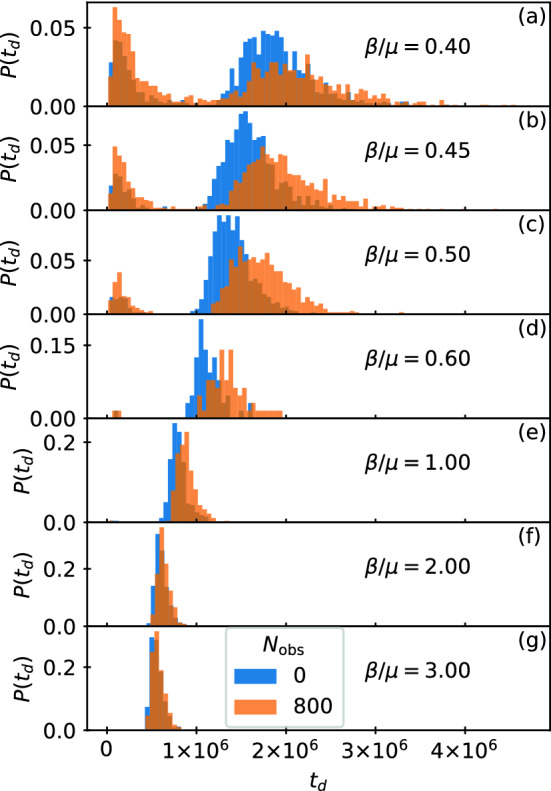
Duration of epidemics with and without quenched disorder in the low and high transmissibility regimes. Distribution $P(t_d)$ of the duration $t_d$ of the epidemic in simulation time steps for 1000 realizations. Blue curves are for a system with no obstacles and orange curves are for a system with obstacles. The low transmissibility regime is $\beta /\mu =$ (**a**) 0.4, (**b**) 0.45, (**c**) 0.5, (**d**) 0.6, and (**e**) 1.0, while the high transmissibility regime is $\beta /\mu =$ (**f**) 2.0 and (**g**) 3.0. The distributions (**a–e**) in the low transmissibility regime are bimodal, and the addition of quenched disorder increases the number of failed outbreaks and increases the duration of successful epidemics. In the high transmissibility regime (**f,g**), there are no failed outbreaks and the effect of quenched disorder is strongly reduced.

### Duration of epidemic

Our simulations reveal a strong stochasticity of the behavior. Depending on the particular randomly chosen locations of the index cases, the duration $t_d$ of the epidemic can vary widely. In particular, for some realizations the outbreak fails to take hold and is extinguished without affecting a significant fraction of the particles. To illustrate this, in Fig. 6 we plot the distribution $P(t_d)$ of the epidemics measured in simulation time steps with and without obstacles for 1000 realizations. In Fig. 6a–e, we show the low transmissibility regime with $\beta /\mu =0.4, 0.45$, 0.5, 0.6, and 1.0. Here the distribution is bimodal and there is a clear division between small $t_d$, where we find failed outbreaks that do not infect a significant fraction of the particles, and larger $t_d$, where successful epidemics occur that involve a substantial fraction of the particles. This behavior is similar to what has been observed in other studies^41^. Addition of quenched disorder in this regime increases the probability that the outbreak will fail, but also increases the average duration of successful epidemics. In contrast, for high transmissibility, as shown in Fig. 6f,g at $\beta /\mu = 2.0$ and 3.0, $P(t_d)$ is unimodal since all outbreaks produce successful epidemics. Additionally, there is no longer a significant difference in the distribution for systems with and without quenched disorder.

**Figure 7:**
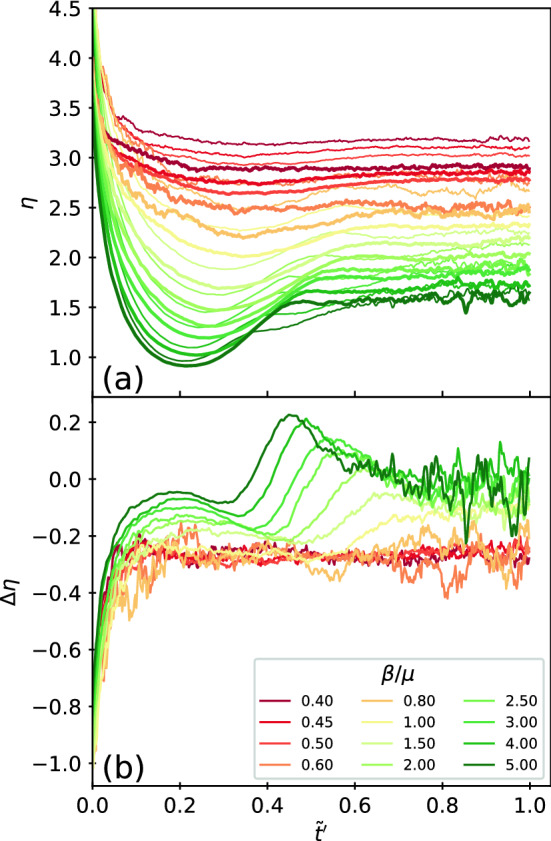
Measure of the ability of I to contact S and how it changes when quenched disorder is introduced. (**a**) $\eta$ vs $\tilde{t}^\prime$ for varied $\beta /\mu$ in samples without obstacles (thin lines) and with obstacles (thick lines). When $\beta /\mu$ is large, a local minimum in $\eta$ appears near $\tilde{t}^\prime =0.1$ due to the formation of a propagating front. (**b**) The difference $\Delta \eta$ between the value of $\eta$ in the sample with disorder and the value in the sample without disorder as a function of $\tilde{t}^\prime$. For $\beta /\mu \le 1.5$, there is no front propagation and the addition of quenched disorder always reduces the value of $\eta$. For $\beta /\mu > 1.5$, a front appears, and once the front has passed, $\Delta \eta$ drops below zero, indicating an enhancement of the infection rate when quenched disorder is present.

### Ability of I to contact S

We can also distinguish the two regimes of behavior using features in $\eta$ by comparing the value of $\eta$ in samples with and without quenched disorder. In Fig. 7a we plot $\eta$ versus $\tilde{t}^\prime$ in samples with and without obstacles. The time scale $\tilde{t}^{\prime }$ reaches a value of $\tilde{t}^{\prime }=1.0$ when the number of recovered has increased to 95% of its maximum value, $r(\tilde{t}^\prime =1.0)=0.95r_{\infty }=0.95(1-s_{\infty })$. Use of this time scale allows us to exclude the stochastic late time behavior when the last few straggling infectives are recovering. At $\tilde{t}^\prime = 0$, $\eta$ is always high since the initial seed *I* particles are surrounded only by susceptible particles. As the epidemic spreads, the average number of *S* particles around *I* particles decreases. When $\beta /\mu \le 1.5$, the curves monotonically decrease to a saturation value between $\eta =2.5$ to $\eta =3.25$, and samples containing obstacles show lower values of $\eta$. For $\beta /\mu > 1.5$, the epidemic spreads in the form of a front, which is visible as the appearance of a local dip in $\eta$ centered near $\tilde{t}^\prime =0.2$. As the front moves rapidly through the largest cluster, most of the infected particles are surrounded by *I* particles behind the expanding front, while only *I* particles at the edge of the front are adjacent to *S* particles. This depresses the value of $\eta$. Once the front has passed through the cluster, the mobility of the particles bring more *S* from the gas phase into contact with the remaining *I*, and $\eta$ recovers somewhat before saturating to a low value between $\eta =1.5$ and 2.0.

In Fig. 7b we plot the difference $\Delta \eta =\eta _{{\rm obs}=800}-\eta _{{\rm obs}=0}$ between $\eta$ for the samples with and without quenched disorder from Fig. 7a. When $\beta /\mu \le 1.5$, $\Delta \eta$ reaches a constant value and is always negative, $\Delta \eta \approx -0.25$, indicating that the quenched disorder is always reducing the effectiveness of the spread of the epidemic. Once the system enters the front propagation regime for $\beta /\mu > 1.5$, $\Delta \eta$ becomes nonmonotonic and shows local peaks and dips. For $\tilde{t}^\prime <0.4$, there is a dip when the front is passing through the largest clusters. In this regime, $\Delta \eta$ is negative, indicating that the quenched disorder slows the front to some extent. For times above the minimum of the dip, $\Delta \eta$ increases and becomes positive, indicating that the addition of quenched disorder is actually increasing the effectiveness of the epidemic spread. This can also be seen in Fig. 5a, where *i* is reduced in the presence of quenched disorder for $\tilde{t} < 0.175$ but is slightly increased for $\tilde{t} > 0.175$, indicating that the disorder can accelerate the infection at later times. This enhancement of the epidemic arises after the largest cluster has become fully infected and some of the infected particles break away from the cluster and enter the gas phase. Within the gas phase, the quenched disorder induces the formation of smaller localized clusters, as shown in Fig. 1. These smaller clusters, once contacted by an infective, undergo the same rapid front propagation as the initial infection wave. When quenched disorder is not present, there are no smaller clusters and the infection must propagate through the gas phase and infect the remaining *S* particles one by one, an inefficient process.

**Figure 8:**
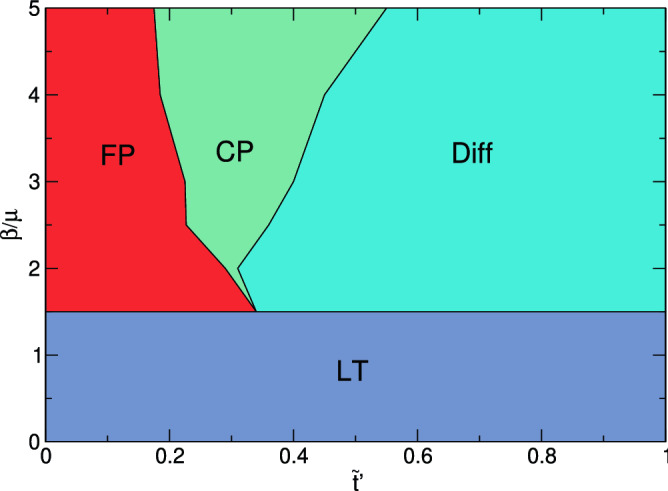
Phase diagram of the epidemic evolution in the low and high transmissibility regimes. The phase diagram of the different regimes as a function of transmissibility $\beta /\mu$ vs reduced time $\tilde{t}^\prime$ based on the features of the curves in Fig. 7 . For $\beta /\mu \le 1.5$, the system is in a low transmissibility (LT) regime where $s_{\infty }>0$ and the addition of obstacles can strongly impact the propagation of the epidemic. The front propagation phase at high transmissibility is marked FP, and in the secondary cluster phase (CP), the addition of obstacles actually increases the spread of the infection. In the diffusive regime (Diff), the obstacles do not affect the epidemic spread.

### Epidemic phase diagram

Based on the features in Fig. 7, we can construct a phase diagram of the behavior of the system as a function of $\beta /\mu$ versus $\tilde{t}^\prime$, as shown in Fig. 8. When $\beta /\mu > 1.5$, $s_{\infty }=0$ and the entire system becomes infected, while the initial invasion of the infection occurs by front propagation. For $\beta /\mu \le 1.5$, the low transmissibility regime marked LT, the infection spreads much more homogeneously, as illustrated in Fig. 2, and $s_{\infty }>0$ so that not all of the particles have been infected by the end of the epidemic. Within this regime, the addition of quenched disorder always reduces $i_{\rm max}$ and increases $s_{\infty }$. In the high transmissibility regime with $\beta /\mu > 1.5$, at small $\tilde{t}^\prime$ the infection propagates as a front through the largest cluster, defined as the front propagation regime FP. Here the addition of quenched disorder can slow the front propagation but does not stop it. After the front has crossed the entire largest cluster, we find the CP regime in which the secondary clusters start to show front propagation. Here the quenched disorder can increase the effectiveness of the spread of the infection by increasing the number of secondary clusters that are present. At larger values of $\tilde{t}^\prime$, all of the clusters have been infected and the epidemic is making its way through the gas phase. In this regime, which we call Diff for diffusive, there is little difference between the systems with and without quenched disorder. The locations of the phase boundaries should depend on the amount of quenched disorder and the activity level of the particles.

**Figure 9:**
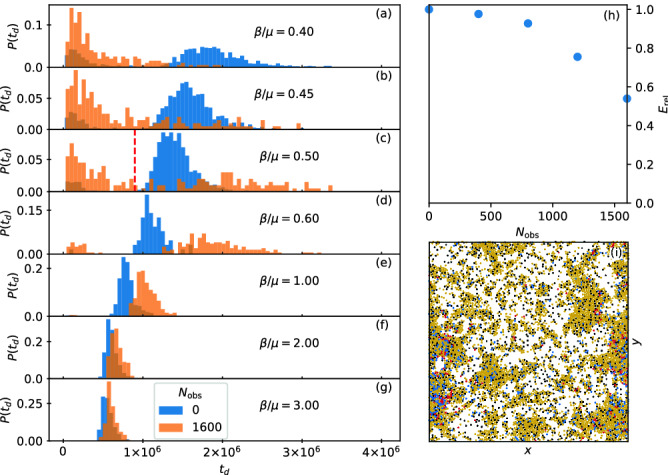
Effect of changing quenched disorder density. (**a–g**) Distribution $P(t_d)$ of the duration $t_d$ of the epidemic in simulation time steps for 200 realizations. Blue curves are for a system with no obstacles and orange curves are for a system with $N_{\rm obs}=1600$ obstacles. The low transmissibility regime is $\beta /\mu =$ (**a**) 0.4, (**b**) 0.45, (**c**) 0.5, (**d**) 0.6, and (**e**) 1.0, while the high transmissibility regime is $\beta /\mu =$ (f) 2.0 and (**g**) 3.0. As was the case for fewer obstacles, in the high transmissibility regime there are no failed outbreaks and the effect of quenched disorder is strongly reduced. (**h**) Relative fraction $E_{\rm rel}$ of successful outbreaks in the presence of quenched disorder compared to the absence of quenched disorder for systems with different numbers of obstacles $N_{\rm obs}$ at $\beta /\mu =0.5.$ The dashed line in panel (**c**) indicates the location of the division between failed and successful outbreaks used in calculating $E_{\rm rel}$. (**i**) Snapshot image of the epidemic for a system with $N_{\rm obs}=1600$ and $\beta /\mu =0.5$ at the peak of the infection showing susceptible (*S*, yellow), infected (*I*, red), and recovered (*R*, blue) states. The system has broken up into a larger number of smaller clusters due to the increased density of quenched disorder sites.

### Effect of changing quenched disorder density

To test the robustness of our results against changes in the density of quenched disorder sites, in Fig. 9a–g we show the distribution $P(t_d)$ of the epidemic durations in a sample with $N_{\rm obs}=1600$ obstacles. In the low transmissibility regime, successful outbreaks are further suppressed as the density of obstacles increases since the large clusters that form for low obstacle density are broken apart at higher obstacle density, as illustrated in Fig. 9i. In the high transmissibility regime of $\beta /\mu \ge 2$, however, even the larger number of quenched disorder sites still has a negligible effect on outbreak duration. Thus our observation that quenched disorder becomes irrelevant for high transmissibility remains robust as the number of obstacles increases.

To quantify the impact of quenched disorder in the low transmissibility regime for the samples with $\beta /\mu =0.5$, we integrate the total number of outbreaks $E_{s}$ in the successful window, defined to be outbreaks with $t_d$ that falls above the red dashed line in Fig. 9c. We then obtain the relative fraction of successful outbreaks, $E_{\rm rel}=E_s^\mathrm{obs}/E_s^0$, where $E_s^\mathrm{obs}$ is the number of outbreaks in samples with quenched disorder and $E_s^0$ is the number of outbreaks in samples without quenched disorder. In Fig 9h the plot of $E_{\rm rel}$ versus $N_{\rm obs}$ indicates that as $N_{\rm obs}$ increases, there is a greater suppression of successful outbreaks compared to the disorder-free system. If the obstacle density became so high that the obstacles themselves begin to percolate across the sample, cutting it into disconnected regions, $E_{\rm rel}$ could drop to zero and different behavior would emerge, but this regime will be studied in a future work.

## Discussion

Our model suggests that active matter can be used to capture a variety of epidemic behaviors. There are a number of active matter systems, such as active colloids, in which the activity of the particles can be controlled on an individual basis using optical rastering methods. Experiments of this type have been developed in order to use active colloids to mimic group formation, to introduce an effective visual perception mobility, and to produce other kinds of collective behaviors such as quorum sensing^31,32^. In order to implement an S-I-R model, individual active colloids could be traced and tagged according to their infective state, and when they interact with other colloids, there can be a probability that the infection will pass to a susceptible colloid. This can be done in a motility induced phase separated regime or in a diffusive regime for varied $\beta /\mu$. The experiments could then be repeated multiple times to obtain the average behavior. Within a given sample, certain colloids could remain inactive and be counted as passive or obstacle particles, or actual obstacles could be put in place on the substrate. Additionally, a wealth of rules could be introduced, such as the inclusion of hyperactive particles that could serve as superspreaders, as well as possible mitigation effects. This could potentially position active matter as a table-top experimental system for modeling epidemics. Our work indicates that active matter can be used as a simulation tool to study epidemics in a system that can be tuned readily between states that are sensitive to spatial disorder and states that are insensitive to disorder.

In conclusion, we have shown how an active matter system of self-propelled particles can be used to model spatial heterogeneity in an S-I-R epidemic spreading model. The active particles naturally form spatial clusters in the motility induced phase separated regime. For low transmissibility, the epidemic spread is percolative and the system is sensitive to the addition of quenched disorder, which both increases the probability of a failed outbreak and increases the average duration of successful epidemics. In this regime, the mixing hypothesis of classical S-I-R models breaks down. For high transmissibility, all of the particles are eventually infected and the epidemic spreads in well defined fronts. In this case, the addition of quenched disorder can slow the spread of the epidemic at early times by slowing the propagation of the initial front. At later times, however, since the quenched disorder introduces a larger number of small clusters in the gas phase, the epidemic can spread more efficiently compared to a system without quenched disorder. Our results indicate that spatial disorder can impact epidemic spreading in both the high and low transmissibility regimes. Our system could be realized experimentally using light activated colloidal particles with specified feedback rules to mimic the S-I-R model, and our results suggest that active matter systems could provide a new way to create table-top epidemic experiments.

## Supplementary Information

Supplementary Figures.

Supplementary Video S1.

Supplementary Video S2.

Supplementary Video S3.

Supplementary Video S4.

## Data Availability

The datasets generated during and/or analyzed during the current study are available from the corresponding author upon reasonable request.

## Code availability

The codes generated and used during the current study are available from the corresponding author on reasonable request.
