# Supplementary material for: Using active matter to introduce spatial heterogeneity to the susceptible infected recovered model of epidemic spreading
Source: Sci Rep. 2022 Jul 4;12:11229. doi: 10.1038/s41598-022-15223-5 (PMC9253087; doi:10.1038/s41598-022-15223-5)
Supplement: Supplementary file 1 — Supplementary Figures. [file 41598_2022_15223_MOESM1_ESM.pdf]

Supplemental material for "Using Active Matter to Introduce Spatial Heterogeneity to the Susceptible Infected Recovered Model of Epidemic Spreading"

P. Forgács,[1] A. Libál,[1] C. Reichhardt,[2] N. Hengartner,[2] and C. J. O. Reichhardt*[2]

[1] *Mathematics and Computer Science Department, Babeş-Bolyai University, Cluj-Napoca 400084, Romania*
[2] *Theoretical Division and Center for Nonlinear Studies, Los Alamos National Laboratory, Los Alamos, New Mexico 87545, USA*

(Dated: June 8, 2022)

Here we present the full epidemic curves of susceptible $s(\tilde{t})$, infected $i(\tilde{t})$, and recovered $r(\tilde{t})$ along with the corresponding $\eta(\tilde{t})$, the average number of S particles surrounding an I particle, for additional values of β/μ that were not included in the main text. Each curve is averaged over 1000 realizations. At $\tilde{t} = 1.0$, the epidemic is over and $i = 0$.

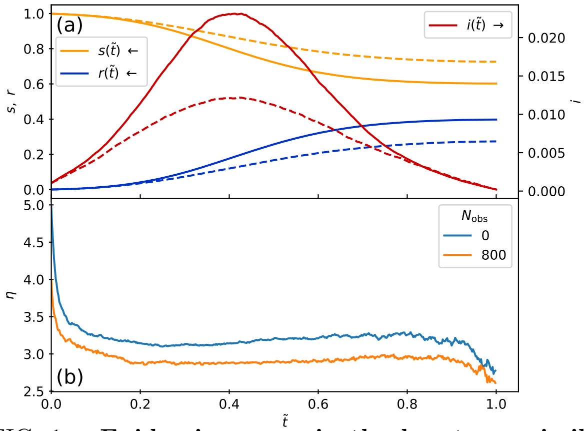

FIG. 1. **Epidemic curves in the low transmissibility regime.** (a) $s(\tilde{t})$ (yellow), $i(\tilde{t})$ (red), and $r(\tilde{t})$ (blue) for a system with $\beta/\mu = 0.4$. Solid lines: no quenched disorder; dashed lines: samples containing obstacles. (b) The corresponding $\eta(\tilde{t})$ without (blue) and with (orange) obstacles.

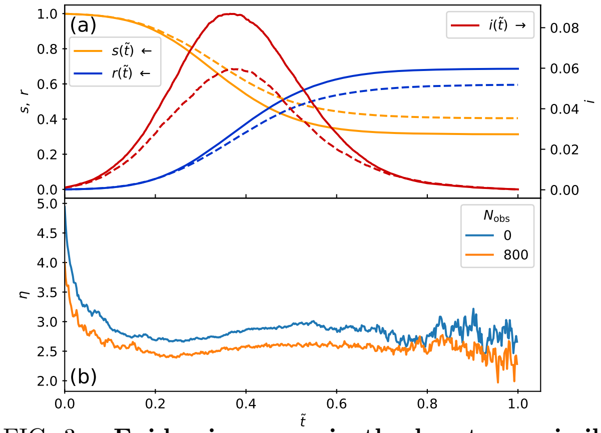

FIG. 3. **Epidemic curves in the low transmissibility regime.** (a) $s(\tilde{t})$ (yellow), $i(\tilde{t})$ (red), and $r(\tilde{t})$ (blue) for a system with $\beta/\mu = 0.6$. Solid lines: no quenched disorder; dashed lines: samples containing obstacles. (b) The corresponding $\eta(\tilde{t})$ without (blue) and with (orange) obstacles.

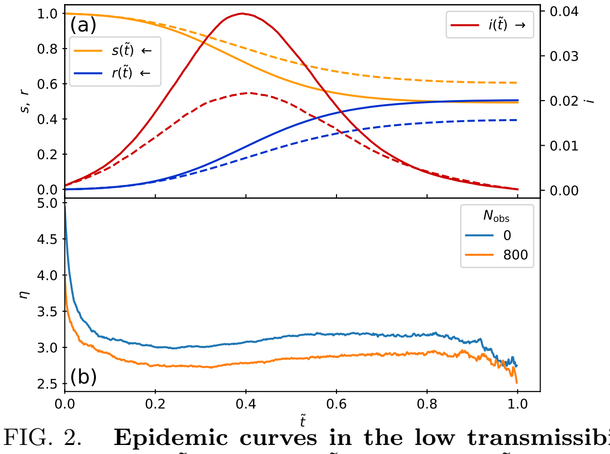

FIG. 2. **Epidemic curves in the low transmissibility regime.** (a) $s(\tilde{t})$ (yellow), $i(\tilde{t})$ (red), and $r(\tilde{t})$ (blue) for a system with $\beta/\mu = 0.45$. Solid lines: no quenched disorder; dashed lines: samples containing obstacles. (b) The corresponding $\eta(\tilde{t})$ without (blue) and with (orange) obstacles.

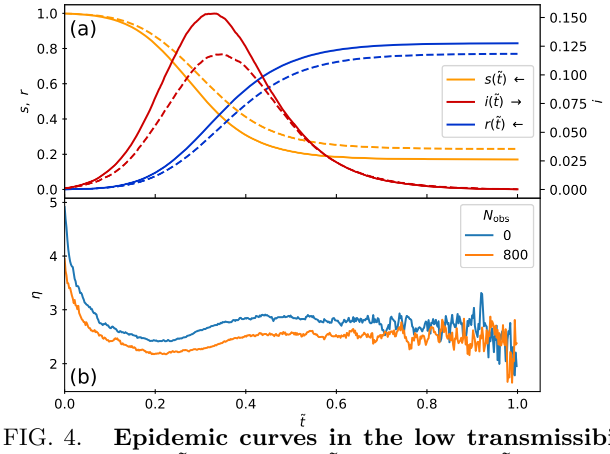

FIG. 4. **Epidemic curves in the low transmissibility regime.** (a) $s(\tilde{t})$ (yellow), $i(\tilde{t})$ (red), and $r(\tilde{t})$ (blue) for a system with $\beta/\mu = 0.8$. Solid lines: no quenched disorder; dashed lines: samples containing obstacles. (b) The corresponding $\eta(\tilde{t})$ without (blue) and with (orange) obstacles.

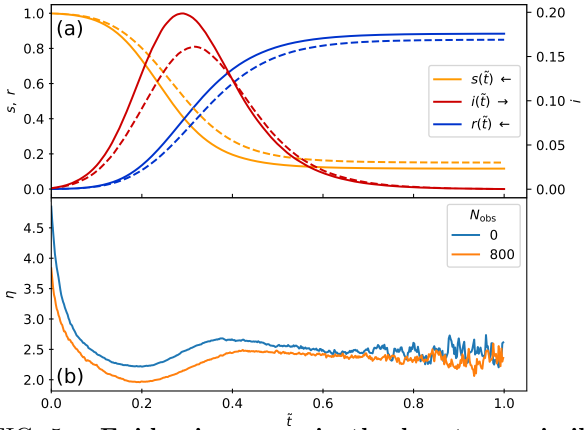

FIG. 5. **Epidemic curves in the low transmissibility regime.** (a) $s(\tilde{t})$ (yellow), $i(\tilde{t})$ (red), and $r(\tilde{t})$ (blue) for a system with $\beta/\mu = 1.0$. Solid lines: no quenched disorder; dashed lines: samples containing obstacles. (b) The corresponding $\eta(\tilde{t})$ without (blue) and with (orange) obstacles.

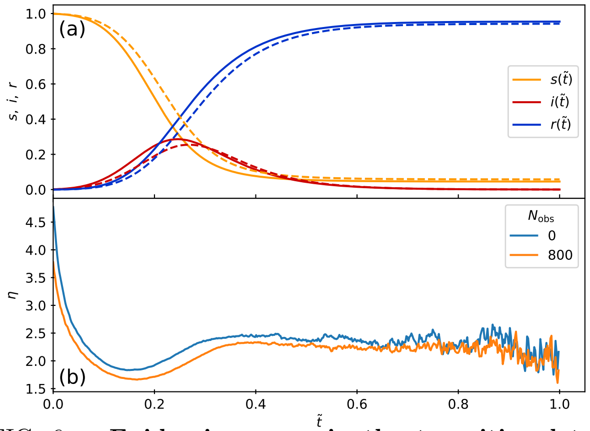

FIG. 6. **Epidemic curves in the transitional transmissibility regime.** (a) $s(\tilde{t})$ (yellow), $i(\tilde{t})$ (red), and $r(\tilde{t})$ (blue) for a system with $\beta/\mu = 1.5$. Solid lines: no quenched disorder; dashed lines: samples containing obstacles. (b) The corresponding $\eta(\tilde{t})$ without (blue) and with (orange) obstacles.

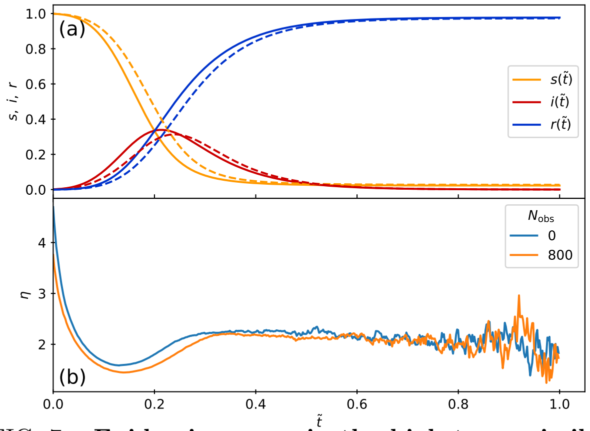

FIG. 7. **Epidemic curves in the high transmissibility regime.** (a) $s(\tilde{t})$ (yellow), $i(\tilde{t})$ (red), and $r(\tilde{t})$ (blue) for a system with $\beta/\mu = 2.0$. Solid lines: no quenched disorder; dashed lines: samples containing obstacles. (b) The corresponding $\eta(\tilde{t})$ without (blue) and with (orange) obstacles.

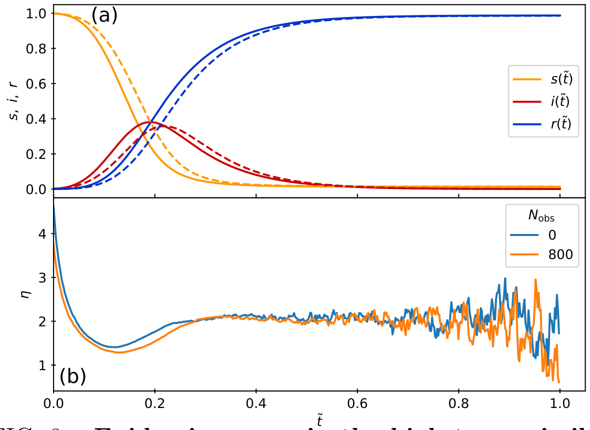

FIG. 8. **Epidemic curves in the high transmissibility regime.** (a) $s(\tilde{t})$ (yellow), $i(\tilde{t})$ (red), and $r(\tilde{t})$ (blue) for a system with $\beta/\mu = 2.5$. Solid lines: no quenched disorder; dashed lines: samples containing obstacles. (b) The corresponding $\eta(\tilde{t})$ without (blue) and with (orange) obstacles.

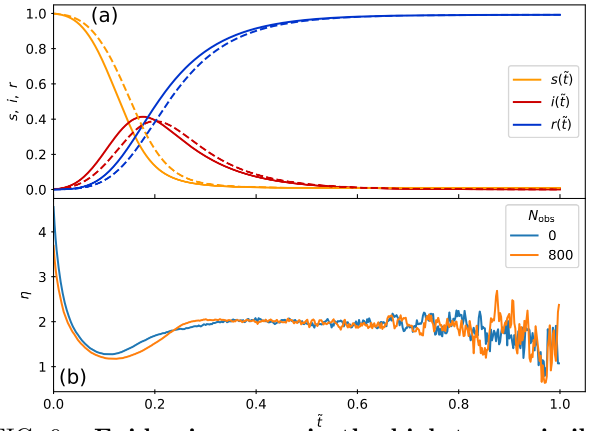

FIG. 9. **Epidemic curves in the high transmissibility regime.** (a) $s(\tilde{t})$ (yellow), $i(\tilde{t})$ (red), and $r(\tilde{t})$ (blue) for a system with $\beta/\mu = 3.0$. Solid lines: no quenched disorder; dashed lines: samples containing obstacles. (b) The corresponding $\eta(\tilde{t})$ without (blue) and with (orange) obstacles.

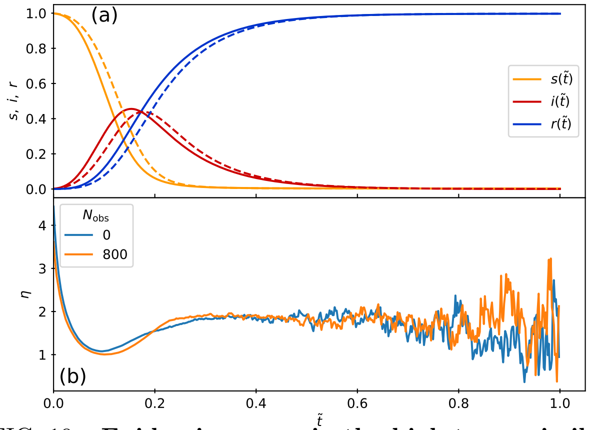

FIG. 10. **Epidemic curves in the high transmissibility regime.** (a) $s(\tilde{t})$ (yellow), $i(\tilde{t})$ (red), and $r(\tilde{t})$ (blue) for a system with $\beta/\mu = 4.0$. Solid lines: no quenched disorder; dashed lines: samples containing obstacles. (b) The corresponding $\eta(\tilde{t})$ without (blue) and with (orange) obstacles.
